# *P. gingivalis* in oral-prostate axis exacerbates benign prostatic hyperplasia via IL-6/IL-6R pathway

**DOI:** 10.1186/s40779-024-00533-8

**Published:** 2024-05-20

**Authors:** Shuang-Ying Wang, Yi Cai, Xiao Hu, Fei Li, Xin-Hang Qian, Ling-Yun Xia, Bo Gao, Lan Wu, Wen-Zhong Xie, Jia-Min Gu, Tong Deng, Cong Zhu, Hai-Chang Jia, Wan-Qi Peng, Jiao Huang, Cheng Fang, Xian-Tao Zeng

**Affiliations:** 1 Center for Evidence-Based and Translational Medicine, Zhongnan Hospital of Wuhan University, Wuhan, 430071 China; 2 Department of Urology, Zhongnan Hospital of Wuhan University, Wuhan, 430071 China; 3 Department of Urology, Huaihe Hospital of Henan University, Kaifeng, 475000 Henan China; 4 Department of Stomatology, Taihe Hospital, Hubei University of Medicine, Shiyan, 442000 Hubei China; 5 Department of Laboratory Medicine, Taihe Hospital, Hubei University of Medicine, Shiyan, 442000 Hubei China; 6 Department of Stomatology, Zhongnan Hospital of Wuhan University, Wuhan, 430071 China; 7 Henan Provincial Engineering Research Center for Microecological Regulatory of Oral Environment and Oral Implantology, Kaifeng University Health Science Center, Kaifeng, 475000 Henan China; 8 School of Stomatology, Jinan University, Guangzhou, 510632 China

**Keywords:** *Porphyromonas gingivalis*, Benign prostatic hyperplasia, Periodontitis, Oral pathogens, Inflammation

## Abstract

**Background:**

Benign prostatic hyperplasia (BPH) is the most common disease in elderly men. There is increasing evidence that periodontitis increases the risk of BPH, but the specific mechanism remains unclear. This study aimed to explore the role and mechanism of the key periodontal pathogen *Porphyromonas gingivalis* (*P. gingivalis*) in the development of BPH.

**Methods:**

The subgingival plaque (Sp) and prostatic fluid (Pf) of patients with BPH concurrent periodontitis were extracted and cultured for 16S rDNA sequencing. Ligature-induced periodontitis, testosterone-induced BPH and the composite models in rats were established. The *P. gingivalis* and its toxic factor *P. gingivalis* lipopolysaccharide (*P.g*-LPS) were injected into the ventral lobe of prostate in rats to simulate its colonization of prostate. *P.g*-LPS was used to construct the prostate cell infection model for mechanism exploration.

**Results:**

*P. gingivalis*, *Streptococcus oralis*, *Capnocytophaga ochracea* and other oral pathogens were simultaneously detected in the Pf and Sp of patients with BPH concurrent periodontitis, and the average relative abundance of *P. gingivalis* was found to be the highest. *P. gingivalis* was detected in both Pf and Sp in 62.5% of patients. Simultaneous periodontitis and BPH synergistically aggravated prostate histological changes. *P. gingivalis* and *P.g*-LPS infection could induce obvious hyperplasia of the prostate epithelium and stroma (epithelial thickness was 2.97- and 3.08-fold that of control group, respectively), and increase of collagen fibrosis (3.81- and 5.02-fold that of control group, respectively). *P. gingivalis* infection promoted prostate cell proliferation, inhibited apoptosis, and upregulated the expression of inflammatory cytokines interleukin-6 (IL-6; 4.47-fold), interleukin-6 receptor-α (IL-6Rα; 5.74-fold) and glycoprotein 130 (gp130; 4.47-fold) in prostatic tissue. *P.g*-LPS could significantly inhibit cell apoptosis, promote mitosis and proliferation of cells. *P.g*-LPS activates the Akt pathway through IL-6/IL-6Rα/gp130 complex, which destroys the imbalance between proliferation and apoptosis of prostate cells, induces BPH.

**Conclusion:**

*P. gingivalis* was abundant in the Pf of patients with BPH concurrent periodontitis. *P. gingivalis* infection can promote BPH, which may affect the progression of BPH via inflammation and the Akt signaling pathway.

**Supplementary Information:**

The online version contains supplementary material available at 10.1186/s40779-024-00533-8.

## Background

Benign prostatic hyperplasia (BPH) is the leading cause of work disability in men over 55 years old worldwide [1]. There were 112.65 × 10^5^ cases of BPH in 2019, an increase of 105.70% over 1990 [2]. The long disease course, poor efficacy and heavy disease burden have seriously affected the quality of life of male patients worldwide [3]. BPH is a complex inflammatory disease, and a study has shown that inflammation plays a key role in the etiology of BPH [4]. Periodontitis is a chronic inflammatory disease, caused by destructive dental pathogens, has become a worldwide challenge [5–8]. Epidemiological study has shown that periodontitis significantly increases the risk of BPH after adjusting for confounding factors [9], and other studies have found a significant correlation between prostate-specific antigen (PSA) concentration and periodontal disease-related indicators [10, 11]. In addition, our previous study of 2171 Chinese men found a significant positive association between periodontal disease and an increased risk of BPH [12]. Our previous study demonstrated that periodontitis might exacerbate or promote BPH through regulation of oxidative stress and the inflammatory process [13]. We have also summarized the findings related to periodontal diseases and prostatic diseases, focusing on the potential molecular mechanism of oral microbiome in these diseases to provide potential therapeutic targets [14]. The above studies indicate that periodontitis may promote BPH and the presence of an oral-prostate axis, and oral pathogens may be involved in the pathogenesis of prostate disease. However, the specific role of these bacteria remains to be elucidated, and they may potentially be developed as new biomarkers for BPH.

Periodontitis is one of the most prevalent infectious diseases affecting oral health globally, and good oral health is crucial for enhancing life quality since it serves as the gateway to general health [15, 16]. Increasing evidence suggests an independent link between periodontitis and systemic diseases such as diabetes and cardiovascular disease. The possible mechanisms include metastatic infections, distal end migration of oral inflammatory-activated Th17 cells, dissemination of bacterial toxins and metabolic and immune disorders [5, 17–19]. The oral flora is very diverse having the second highest complexity in terms of microorganisms after the large intestine. These regularly balance with the host and protect against pathogenic microorganisms [20, 21]. Studies have shown that oral pathogens can be transferred to non-oral sites through oropharyngeal, oral digestive translocation, blood-derived transmission and other routes to cause infection [5], and bacterial infections could promote the progression of diseases such as gastrointestinal diseases, colorectal cancer, atherosclerosis, prostatic diseases, lung cancer and esophageal cancer [18, 22, 23].

*P. gingivalis*, a species of “red complex”, is known to be a major causative agent of periodontitis [5]. It is reported that *P. gingivalis* can transfer from the oral cavity to pancreas and promote pancreatic cancer progression by increasing the secretion of neutrophil chemokine and neutrophil elastase [24]. Live *P. gingivalis* and its virulence factors can induce inflammation in the brain and directly affect the progression of Alzheimer’s disease [25, 26]. Lipopolysaccharides from *P. gingivalis* (*P.g*-LPS) are identified as the main virulence factor stimulating a wide range of host immune responses and activating multiple cell types via the production of pro-inflammatory cytokines [27]. Estemalik et al. [28] detected *Treponema denticola* and *P. gingivalis* genomic DNA in prostatic fluid (Pf) and dental plaque of 46.7% and 35.3% of patients with prostate disease (BPH and prostatitis) concurrent periodontitis, respectively. Metastatic infection of oral pathogens may be one of the reasons by which periodontitis promotes the progression of BPH, but which oral pathogens are responsible and the underlying mechanisms remain to be studied.

In this study, firstly we identified the specific species of oral pathogens associated with periodontitis concurrent BPH by microbial culture and sequencing in human samples. Then we explored the role of *P. gingivalis* (with the highest relative abundance found in Pf of patients) in BPH development by constructing rat models of ligature-induced experimental periodontitis and direct bacterial infection of prostate. Finally, in vitro experiments on *P.g*-LPS infected WPMY-1 cells were further conducted to investigate the possible mechanism of *P. gingivalis* affecting BPH. The study aimed to explore the mechanism role of the key periodontal pathogen *P. gingivalis* in the development of BPH and provide new insights for prevention and treatment of BPH in the future.

## Methods

### Human subjects and sample collection

All protocols were approved by the Ethics Committee of Zhongnan Hospital of Wuhan University (2019102). The inclusion criteria for this study were adult males with clinical diagnosis of BPH concurrent chronic periodontitis, and the presence of at least 12 natural teeth. Exclusion criteria included patients who had received periodontal-related treatment within the last 3 months and those who had received antibiotic therapy within the first 6 months. Eight subjects were recruited in Zhongnan Hospital of Wuhan University from November 2019 to December 2019, and they all gave informed consent. Pf samples were collected by an experienced urologist performing prostate massage, and subgingival plaque (Sp) samples were collected by a stomatologist [28]. All samples were placed in sterile culture tubes immediately after collection.

### Bacterial DNA extraction

The collected Pf samples and Sp samples were inoculated on Columbia blood agar plates, MacConkey plates and anaerobic blood agar plates. Columbia blood agar plates and MacConkey plates were placed in a 5 – 10% CO_2_ aerobic environment, and anaerobic blood agar plates were placed in an anaerobic environment, and incubated at 35 °C for 18 – 24 h. Then several growing colonies were picked, boiled in 300 μl double-distilled water for 10 min, and centrifuged at 13,000 rpm for 3 min to extract the supernatant and obtain bacterial DNA. DNA concentration and quality were assessed using a NanoDrop spectrophotometer (NanoDrop Technologies, Wilmington, USA).

### Full-length 16S rDNA gene sequencing

The extracted bacterial DNA was amplified by PCR using primers 27F: (5’-AGRGTTTGATYMTGGCTCAG-3’) and 1492R: (5’-GGYTACCTTGTTACGACTT-3’). After purification of the PCR products, SMRTbell adapters were added to construct a library, and the library was sequenced using the PacBio SMRT DNA sequencing system (Frasergen, Wuhan, China). The raw sequencing data was processed using the SMRT Link software (v8.0) to obtain circular consensus sequence (CCS) reads. Then, the CCS reads were subjected to primer sequence removal and length filtering (1300 – 1600 bp) to obtain the final clean reads for subsequent analysis. The raw data have been deposited under NCBI BioProject accession No. PRJNA863440.

### Bioinformatic analysis

In order to analyze the species composition and diversity information of the samples, QIIME software (v1.80) was used to perform operational taxonomic units (OTUs) clustering on the clean reads of all samples with 97% consistency, and chimeras were removed during the clustering process [29]. Using QIIME software, the samples with the lowest data volume were leveled to eliminate the impact of sequencing depth. Subsequently, the Ribosomal Database Project (RDP) classifier Bayesian algorithm with a minimum confidence threshold of 0.8 was used to compare OTU representative sequences with the NCBI 16S ribosomal RNA (Bacteria and Archaea) database for species annotation. OTU-based α-diversity was estimated by calculating Chao1, observed OTUs, Shannon, Simpson and coverage using QIIME software. β-diversity is estimated by calculating Bray-Curtis dissimilarity (vegan package in R 3.1.1) and unweighted UniFrac distance (QIIME, v1.80).

### Animal experiments

Animal studies were performed in strict accordance with the protocol approved by the Animal Ethics Committee of Wuhan University (2019128), and compliant with the ARRIVE guidelines. Seven-week-old adult Sprague-Dawley male rats reared in the Animal Experiment Center of Zhongnan Hospital of Wuhan University were used (*n =* 58). The breeding environment is specified as pathogen-free with a constant temperature (22 – 26 °C) and humidity (40 – 70%). The drinking water and feed are sterilized by high pressure steam. All rats were adaptively fed for 1 week before the experiment.

#### Experimental design 1

The rats were randomly divided into 6 groups (*n* = 5/group): 1) a sham-operated group (sham) undergoing sham operation for castration and subcutaneous injection of normal saline [5 mg/(kg·d)] for 4 weeks; 2) a testosterone-induced BPH group (T-BPH) undergoing castration and subcutaneous injection of testosterone propionate [5 mg/(kg·d)] (Ningbo Second Hormone Factory, Ningbo, China) for 4 weeks [30]; 3) a ligature-induced experimental periodontitis group (EP) undergoing ligation using sterile nylon thread around the cervical of maxillary first and second molars on both sides [5] and subcutaneous injection of normal saline [5 mg/(kg·d)] for 4 weeks; 4) a composite group (EP + BPH) undergoing castration and subcutaneous injection of testosterone propionate as the testosterone-induced BPH group, simultaneously, the maxillary first and second molars of rats were treated the same way as the ligature-induced EP group [13]; 5) *P. gingivalis* induced periodontitis group (*P.g*) undergoing ligation using sterile nylon with *P. gingivalis* (1 × 10^8^ CFU/ml) thread around the cervical of maxillary first and second molars on both sides [5] and subcutaneous injection of normal saline [5 mg/(kg·d)] for 4 weeks; 6) healthy group (healthy) received no intervention. The above surgical procedures were conducted under anesthesia by intraperitoneal injection of sodium pentobarbital (40 mg/kg) and all rats received subcutaneous injections in the week after surgery.

#### Experimental design 2

The rats were randomly divided into 4 groups (*n* = 7/group) using a computer-based random order generator: 1) control group (Control) was injected with 100 μl normal saline with equal amounts into the right and left ventral lobes of the prostate; 2) *P.g*-LPS induced BPH group (LPS-BPH) were injected with 100 μl *P.g*-LPS (100 μg/kg, SMB00610, Sigma, St. Louis, MO, USA) with equal amounts into the right and left ventral lobes of prostate, in accordance with the *Escherichia coli* (*E. coli*)-LPS induced BPH rats model [31, 32]; 3) The *P. gingivalis* induced BPH group (*P.g*-BPH) were injected with 100 μl of *P. gingivalis* (1 × 10^8^ CFU/ml) with equal amounts into the right and left ventral lobes of prostate, in accordance with the direct injection of live *E. coli* or *P. gingivalis* induced rats model [33, 34]; 4) testosterone-induced BPH group (T-BPH) underwent castration and subcutaneous injection of testosterone propionate [5 mg/(kg·d)] for 4 weeks. All rats were fasted for 6 h before the above surgery. During the experiment, we strictly controlled the diet and living environment of the rats. They all lived in the same environment and ate the same food, thus eliminating confounding factors such as diet, obesity and stress.

### Harvest of the rat prostate

After 4 weeks all rats were weighed and euthanized using an overdose of anesthetic. The bladder, seminal vesicle and prostate were completely removed and photographed. Prostate tissues were weighed, subsequently dissected and fixed with 4% paraformaldehyde (Beijing Labgic Technology Co., Ltd., Beijing, China) for histological and immunohistochemical analysis. The prostate weight index was calculated by (prostate weight of each animal/body weight of each animal) × 1000. The statisticians responsible for the randomization process and the animal experimenters are the only ones who know the distribution of the groups, while the staining and serum marker testing personnel and data analysis statisticians were blind to the experimental grouping.

### Micro-computed tomographic (micro-CT) analyses of alveolar bone

Bilateral maxillary alveolar bone samples containing molars were harvested and fixed with 4% paraformaldehyde. The left maxillae were dissected free of soft tissues to evaluate alveolar bone loss in each group by micro-CT (Skyscan 1176, Bruker, Kontich, Belgium). The generator was operated at a source voltage of 85 kV and source current of 200 μA with an exposure time of 384 ms and an image pixel size of 9 µm. Three-dimensional reconstruction was performed by the NRecon software (Bruker, Kontich, Belgium) after scanning. The cement-enamel junction to alveolar bone crest (CEJ-ABC) was measured by DataViewer (Bruker, Kontich, Belgium) for the maxillary second molar of 4 sites as follows: the proximal buccal, proximal palatal, distal buccal and distal palatal. The residual side of the maxillae was subsequently used for histological evaluation.

### Histopathological analyses

The fixed prostate tissue and periodontal tissues were dehydrated in gradient alcohol and embedded in paraffin, and 3-μm thick sections were prepared. Sections were stained with hematoxylin and eosin (HE; Servicebio, Wuhan, China) according to the manufacturer’s instructions, and images were taken using an optical microscope (Leica DFC295, Wetzlar, Germany). Masson’s trichrome staining was performed according to standard procedures, prostatic epithelia, smooth muscle (SM) and collagen fibers (CF) were stained in red, dark red and blue, respectively. The images were observed and photographed with an inverted phase contrast microscope (Leica, Wetzlar, Germany), and the area percentage of each part was quantitatively analyzed using ImageJ software (National Institutes of Health, USA).

### Immunohistochemistry (IHC) staining

Prostate tissue sections (4 μm) were deparaffinized and then treated with EDTA buffer and 3% hydrogen peroxide solution successively for antigen retrieval and elimination of endogenous peroxidase activity. Samples were incubated overnight at 4 °C with 50 μl of the primary antibodies, and followed by incubation with the second antibody for 50 min at 37 °C. The antibodies are described in Additional file 1: Table S1. After color development and counterstaining with DAB solution (Beijing Zhongshan Jinqiao Biotechnology Co., Ltd., Beijing, China) and hematoxylin, respectively, pictures were taken with the MicroPublisher imaging system (QImaging, Canada). All staining was analyzed using ImageJ software.

### Terminal deoxynucleotidyl transferase-mediated dUTP nick end labeling (TUNEL) assay

Prostate tissue sections (4 μm) were deparaffinized and subjected to routine experimental steps such as washing, dilution, labeling, and protein degradation. Then, TUNEL assay was performed using TUNEL apoptosis detection kit (Roche, Basel, Switzerland) according to the manufacturer’s instructions. Ten fields of view were randomly selected, and TUNEL-positive cells (nuclei were stained in green) were identified under a light microscope (×400) and recorded. The apoptosis rate (%) was analyzed by ImageJ software, and the calculation formula was: apoptosis rate (%) = (number of TUNEL positive cells/total number of cells) × 100%.

### Determination of serum markers by enzyme-linked immunosorbent assay (ELISA)

Serum samples were collected from the abdominal aorta before the rats were sacrificed, and separated by centrifugation at 3000 rpm for 15 min. Then serum concentrations of testosterone, estradiol (E_2_) and PSA were measured using a testosterone kit (RK00724, ABclonal Biotechnology, Wuhan, China), a rat E_2_ ELISA kit (ELK8714, ELK Biotechnology, Denver, USA) and a rat PSA ELISA kit (ELK9631, ELK Biotechnology, Denver, USA). The measurement results were analyzed using Curve Expert software, and the function models were used to fit the standard curve and calculate the results.

### Cell culture, reagents, and cell transfection

Human prostate stromal cell line WPMY-1 was kindly provided by Stem Cell Bank, Chinese Academy of Sciences (Shanghai, China), and cultured in DMEM (Hyclone, USA) containing 5% fetal bovine serum (Gibco, USA) with 1% penicillin G sodium/streptomycin sulfate at 37 °C with 5% CO_2_. MK2206, which is a phosphorylated Akt (p-Akt) inhibitor (Beyotime Biotechnology, Shanghai, China). For *IL-6Rα* (interleukin-6 receptor-α) knockdown, siRNAs and the corresponding controls were purchased from the GenePharma (Shanghai, China) and transfected into cells with Lipofectamine 2000 (Invitrogen, CA, USA). The target sequences are shown in Additional file 1: Table S2.

### Cell apoptosis, cycle, and proliferation assay

WPMY-1 cells (2 × 10^5^ cells/well) were seeded into 6-well plates and cultured at 37 °C for 24 h. Then the medium was replaced with DMEM containing 0 μg/ml, 0.1 μg/ml and 1 μg/ml *P.g*-LPS (SMB00610, Sigma, St. Louis, MO, USA), respectively. For cell apoptosis analysis, 1 × 10^6^ cells were collected, washed with PBS, and gently mixed with Annexin V-APC and propyl iodide (PI) staining solution by using the Apoptosis Detection Kit (LiankeBio, Hangzhou, China). It was incubated at room temperature for 15 min and then detected by flow cytometry (NovoCyte 3000, Agilent Technologies, CA, USA). For cell cycle distribution analysis, 1 × 10^6^ cells were collected and detected by Cell Cycle Staining Kit (LiankeBio, Hangzhou, China) according to the manufacturer’s instructions. Then incubated at room temperature for 30 min and detected by flow cytometry. For cell proliferation, cell culture was continued for 0 h, 24 h, 48 h and 72 h. Then 10 μl of Cell Counting Kit-8 assay (CCK-8, Dojindo, Kumamoto, Japan) solution was added to each well, follow the manufacturer’s instructions, and finally the absorbance at 450 nm was measured using a microplate reader (K3 TOUCH, LabServ, United States).

### Immunofluorescence (IF) cell staining

WPMY-1 cells were seeded onto sterile confocal petri dishes (Biosharp, Anhui, China), fixed in methyl alcohol for 15 min, permeabilized by 0.1% Triton X-100, and then blocked with 1% BSA for 30 min. The cells were then incubated with interleukin-6 receptor (IL-6R) and glycoprotein 130 (gp130) antibodies overnight at 4 °C. They were then probed with secondary antibodies for 1 h at room temperature, and finally nuclear counterstaining was performed with DAPI (Beyotime, Shanghai, China). The antibodies are shown in Additional file 1: Table S1. Coverslips were examined with a fluorescence microscope (ZEISS, Germany).

### RNA extraction and reverse transcription‑quantitative PCR (RT‑qPCR)

WPMY-1 cells cultured with DMEM containing 0 μg/ml or 1 μg/ml *P.g*-LPS (SMB00610, Sigma, St. Louis, MO, USA) for 24 h. Total RNA was extracted from cells using an RNA extraction kit (Promega, Beijing, China) according to the manufacturer’s instructions and cDNA was subsequently synthesized using a TaKaRa reverse transcription kit (RR047A, Takara, Japan). qPCR was performed on the Bio-Rad CFX Connect Real-Time System using the SYBR Green kit (TaKaRa, Shiga, Japan). Amplification conditions were 95 °C for 3 min and 40 cycles each at 95 °C for 10 s, 60 °C for 20 s, and 72 °C for 20 s. Primers sequences are shown in Additional file 1: Table S2. Results were normalized to the housekeeping gene GAPDH and relative gene expression levels were calculated using 2^^-(ΔΔCt)^ method.

### Western blotting

Western blotting was performed as described previously [11]. The antibodies used in this study are shown in Additional file 1: Table S3.

### Inflammation cytokines panel assay

WPMY-1 cells were cultured with DMEM containing 0 μg/ml or 1 μg/ml *P.g*-LPS (SMB00610, Sigma, St. Louis, MO, USA) for 2 h, 4 h, 12 h and 24 h. Cell culture supernates were collected and identified by the ABplex Human 4-Plex Custom Panel (RK04333, Abclonal Biotechnology, Wuhan, China), which were flexible bead-based multiplex assays. Briefly, 50 μl cell culture supernates and 5 μl microsphere suspension were added into each well, incubated at 37 °C for 1 h, and magnetically washed once; 50 μl antibody solutions were added, incubated at 37 °C for 0.5 h and washed once. Then 50 μl fluorescein solutions were added and incubated at 37 °C for 15 min avoiding light. Finally, 70 μl of wash buffer was used and detected by Multi-index flow analyzer (ABplex-100, Abclonal Biotechnology, Wuhan, China,).

### Statistical analysis

The 16S rDNA sequencing data were evaluated using the Wilcoxon rank-sum test comparisons by the Wilcox-test package in R (v3.4.1). The in vivo and in vitro experiments data were expressed as the means ± SD. Statistical analysis was performed using SPSS 26.0 software (SPSS, Inc., Chicago, IL, USA). The differences between groups were analyzed with one-way analysis of variance (ANOVA); LSD correction (Homogeneous variance) or Games-Howell (Heterogeneity of variance) was applied for post hoc comparisons. Independent sample *t* test was used to compare the two groups. *P* < 0.05 was considered significant.

## Results

### Oral pathogens in the prostates of patients with BPH concurrent periodontitis

**Fig. 1:**
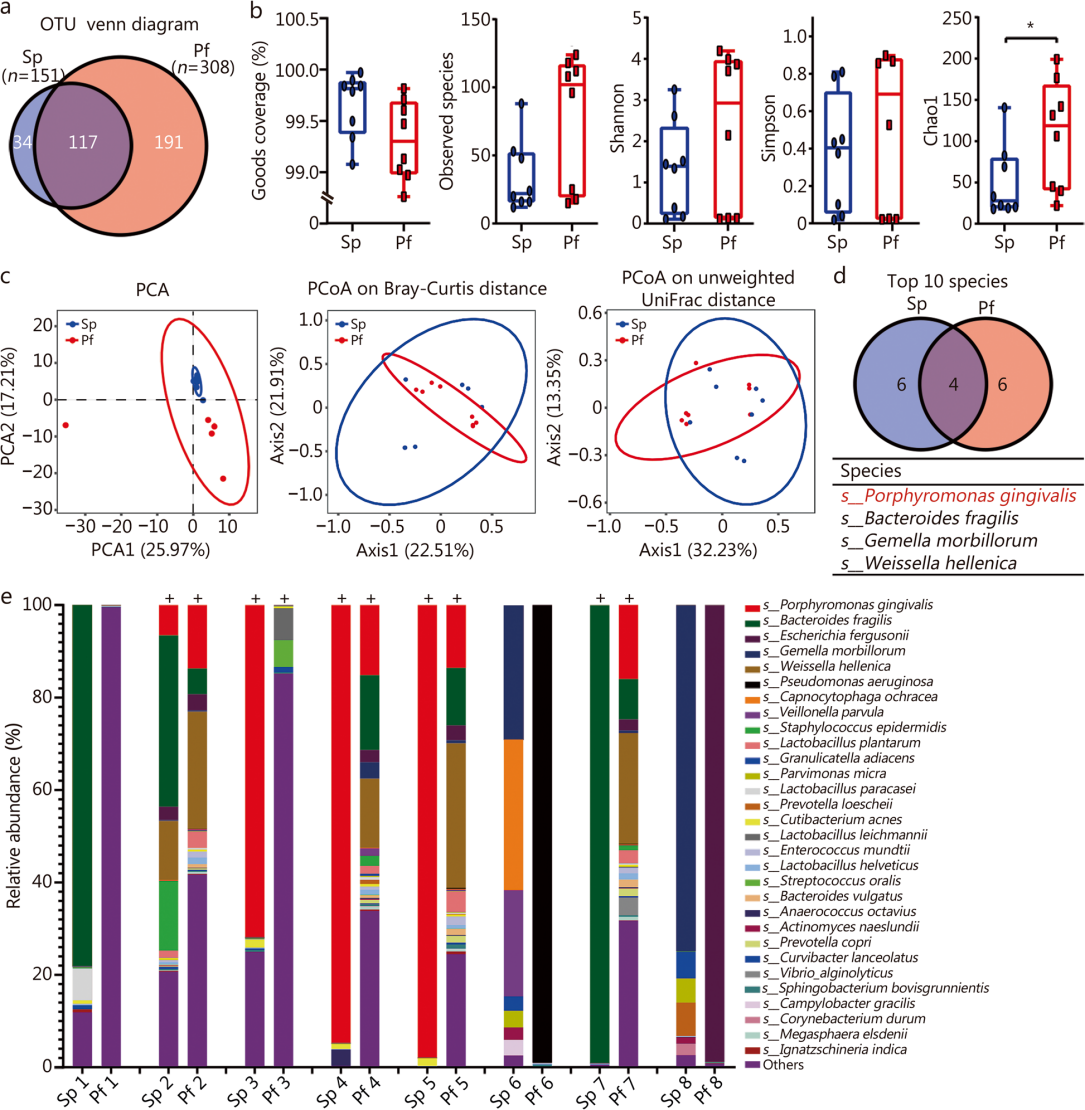
Composition of microbiota community in male patients with BPH concurrent periodontitis. **a** Venn diagram of common and unique OTUs in prostatic fluid (Pf) and subgingival plaque (Sp) samples. **b** α-diversity index of goods coverage, observed species, Shannon, Simpson and Chao 1 in Pf and Sp samples. **c** β-diversity index of principal component analysis (PCA) analysis, principal co-ordinate analysis (PCoA) based on Bray-Curtis distance and Unweighted UniFrac distance matrix in Pf and Sp samples. **d** Venn diagram of commonly detected species in Pf and Sp (the top 10). **e** Relative abundances of microbial composition at the level of species in all samples. Each bar represents a subject sample and each colored box represents a bacterial species. + indicates that *P. gingivalis* was detected. ^*^P < 0.05. OTU operational taxonomy units

A total of 342 OTUs were detected by clustering all the 16s rDNA sequencing clean reads with 97% consistency, of which 117 OTUs were found both in Pf and Sp (Fig. 1a). There was no significant difference in the goods coverage between the two, both reaching more than 98% (Fig. 1b), indicating that the sequencing depth has basically covered all the species in the sample. α-diversity analysis showed that compared with Sp, slightly more species and increased diversity (Shannon and Simpson index) were detected in the Pf samples, but there were no statistical diferences (*P* > 0.05), while community richness (Chao1) was significantly increased (*P* < 0.05, Fig. 1b). β-diversity can reflect the differences in the bacterial community composition in different groups. The results of principal component analysis (PCA) and principal co-ordinate analysis (PCoA) on bray curtis and unweighted UniFrac distance all indicated that Pf and Sp were similar and indistinguishable (Fig. 1c).

To further analyze the microbial composition of Pf and Sp, we classify OTUs by comparing them with the NCBI 16S ribosomal RNA (Bacteria and Archaea) database, and obtained the species composition and relative abundance of each sample. Species ranked after 30th in average abundance were merged into others. The histogram of species composition at the family level showed that *Porphyromonadaceae*, *Bacteroidaceae* and *Streptococcaceae* were the dominant bacteria in 75% of Sp and Pf (Additional file 1: Fig. S1). The venn diagram of the top 10 species in Sp and Pf showed that *P. gingivalis*, *Bacteroides fragiliss*, *Gemella morbillorum* and *Weissella hellenica* were the most abundant species in both samples, and the average abundance of *P. gingivalis* was the highest (Fig. 1d, Additional file 1: Table S4). Composition at species level showed that oral pathogens such as *P. gingivalis*, *Bacteroides fragilis*, *Capnocytophaga ochracea*, *Parvimonas micra* and *Streptococcus oralis* were detected in Pf and Sp (Fig. 1e, Additional file 1: Table S5). *P. gingivalis* was detected in both the Pf and Sp in 62.5% (5/8) of the patients, and accounted for 12 – 15% in Pf in 50% of patients (Fig. 1e). This result is further supported by genus level (Additional file 1: Fig. S2). It is suggested that the keystone periodontal pathogens (especially *P. gingivalis*) may be one of the reasons why periodontitis affects the progression of BPH.

### Experimental periodontitis promoted BPH development

**Fig. 2:**
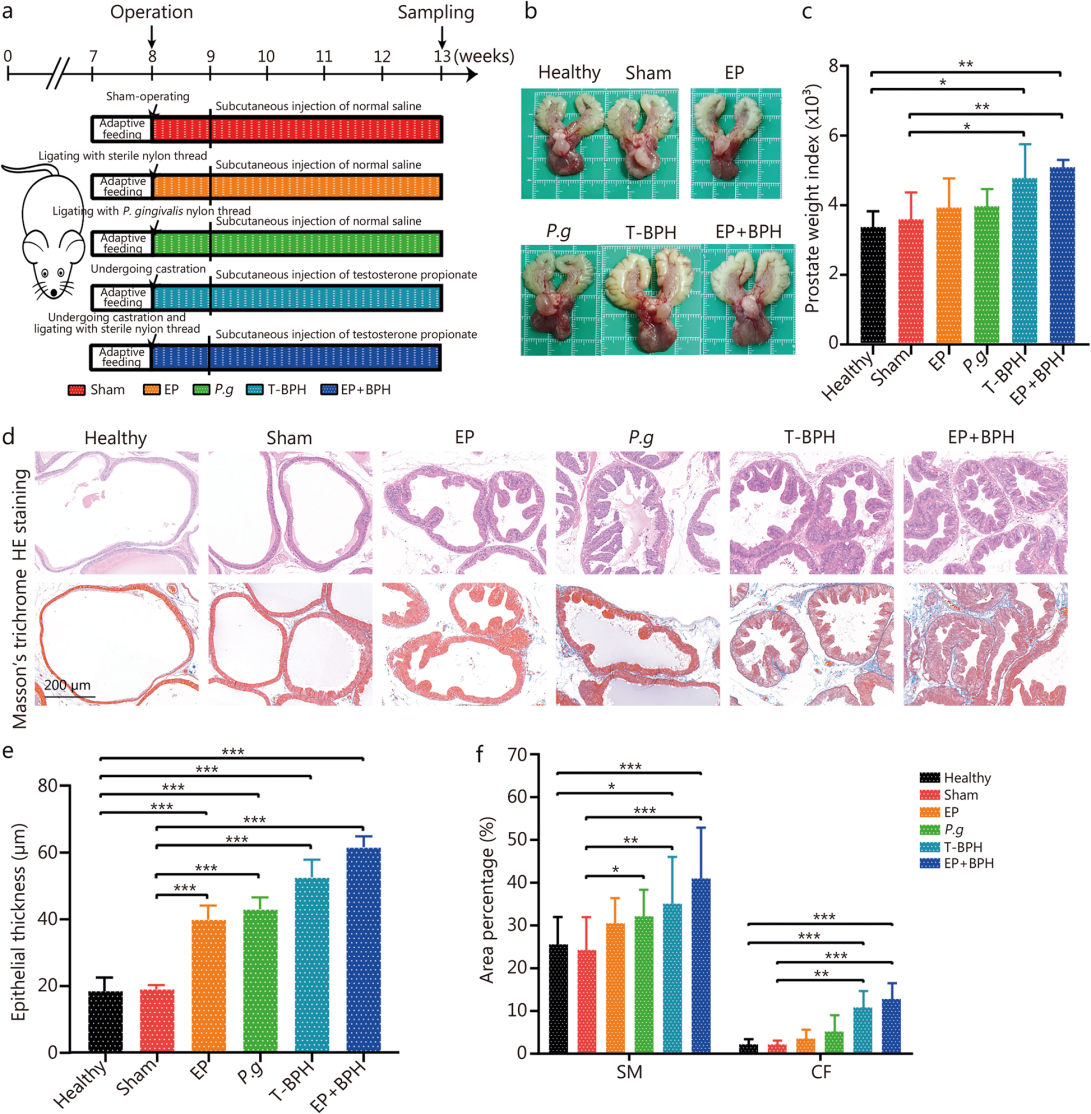
Experimental periodontitis promoted BPH development in rat prostate tissues. **a** Flowchart of experiment periodontitis and BPH rat model establishment. **b** Photographs of ventral prostate, bladder, and seminal vesicle from the healthy, sham, EP, *P.g*, T-BPH and EP + BPH groups. **c** Histogram of prostate weight index (prostate weight of each animal/body weight of each animal) × 1000. **d** Representative figures from HE staining and Masson’s trichrome staining for the prostate tissues. (original magnification ×200). **e** Bar graph for area percentage of epithelia from the groups. **f** Bar graph for area percentage of SM and CF from the groups. Data are expressed as mean ± SD. ^*^P < 0.05, ^**^P < 0.01, ^***^P < 0.001. EP ligature-induced experimental periodontitis group, *P.g Porphyromonas gingivalis* induced BPH group, T-BPH testosterone-induced BPH group, EP + BPH composite group of EP and BPH, HE hematoxylin and eosin, SM smooth muscle, CF collagen fibers

To examine the effect of experimental periodontitis on prostatic hyperplasia, ligature-induced periodontitis (with or without *P. gingivalis*), testosterone-induced BPH and the composite models in rats were established (Fig. 2a). Experimental periodontitis was successfully established in the EP model, *P.g* model and EP + BPH model, confirmed by micro-CT analyses of alveolar bone and histopathologic analyses of periodontal tissues (Additional file 1: Fig. S3a). Statistical analysis also demonstrated that CEJ-ABC distance of the animals in EP, *P.g* and EP + BPH groups was significantly increased when compared with healthy, sham and T-BPH groups (Additional file 1: Fig. S3b). HE staining showed that there was obvious bone resorption, erosion of gingival epithelium with infiltrated inflammatory cell, and loss of epithelial attachment in rats which suffered experimental periodontitis (EP, *P.g* and EP + BPH groups) (Additional file 1: Fig. S3c).

All testosterone-induced BPH (T-BPH and EP + BPH) rats showed significant increase of prostate volume (Fig. 2b) and prostate weight index (Fig. 2c), which are the most visualized feature of BPH pathology. Microscopic examination (HE staining) showed that prostatic tissues obtained from healthy and sham groups rats retained normal structure with numerous acini containing homogenous acidophilic material. Whereas, prostates taken from the ligature-induced periodontitis group and testosterone-induced BPH group, displayed marked glandular hyperplasia and a decreased glandular luminal area compared to the control groups. Masson’s trichrome staining showed that *P.g*, T-BPH and EP + BPH rats displayed obviously increased stromal cells in the prostate (Fig. 2d). Quantitative analysis of histological components showed that compared with healthy and sham groups, the EP and *P.g* groups had increased thickness of the epithelial layer, and the *P.g* group also showed obvious increasing of SM. The T-BPH group showed increased epithelium, SM, and CF; the EP + BPH group demonstrated the most obvious increase in epithelial hyperplasia, SM, and CF (*P* < 0.001; Fig. 2e, f). The observation of pathology changes in the prostate, especially in EP + BPH, indicated an aggravating effect of periodontitis on prostate hyperplasia.

### Transplantation of *P. gingivalis* induced BPH symptoms in rats

**Fig. 3:**
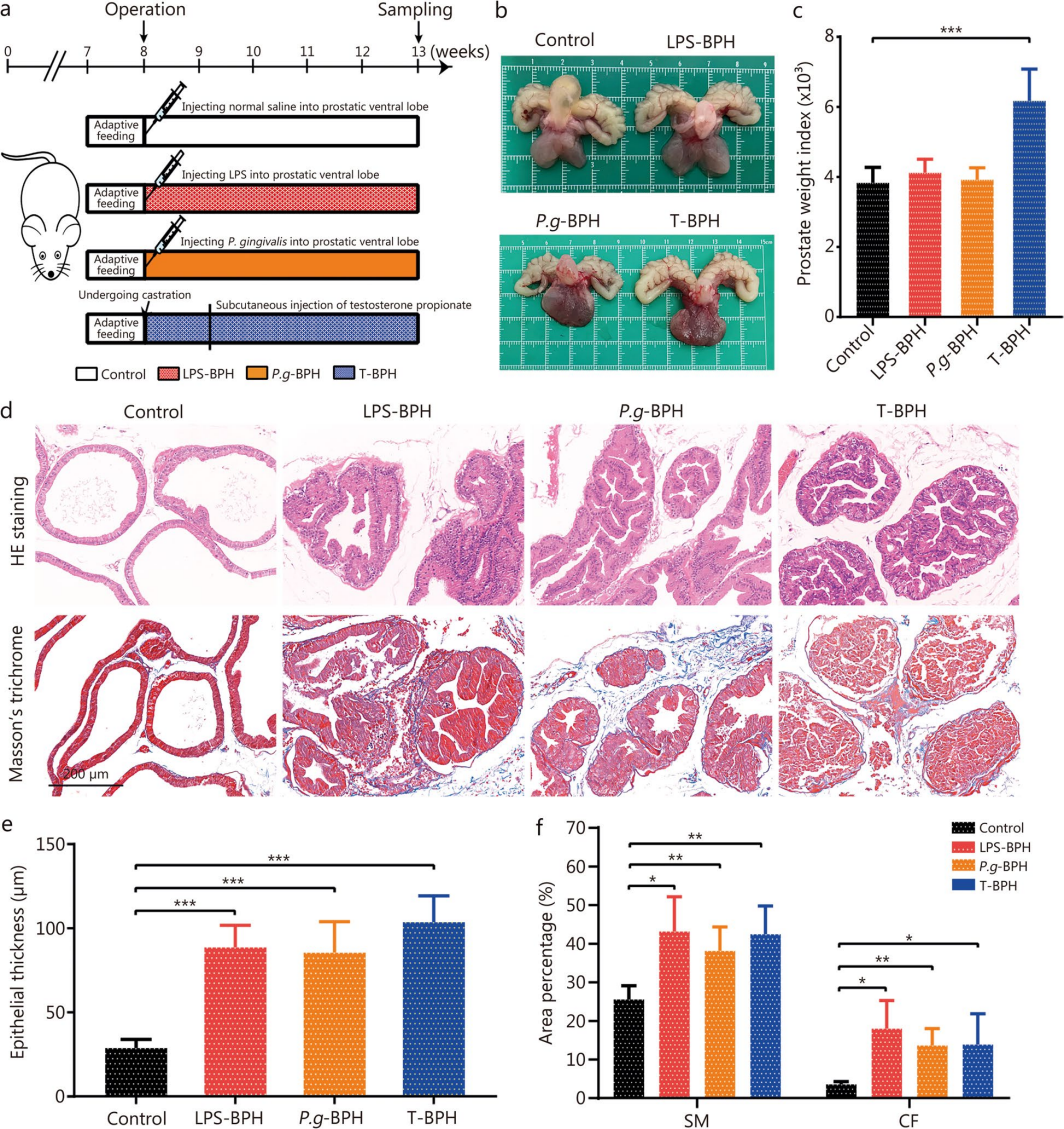
Transplantation of *P. gingivalis* induced BPH symptoms in rat prostate tissues. **a** Flowchart of *P. gingivalis* and *P. gingivalis* LPS infection rat model establishment. **b** Photographs of ventral prostate, bladder, and seminal vesicle from the control, *P.g*-BPH, LPS and T-BPH groups. **c** Histogram of prostate weight index (prostate weight of each animal/body weight of each animal) × 1000. **d** Representative figures from HE staining and Masson’s trichrome staining for the prostate tissues (original magnification ×200). **e** Bar graph for area percentage of epithelia from the groups. **f** Bar graph for area percentage of SM and CF from the groups. Data are expressed as mean ± SD. ^*^P < 0.05, ^**^P < 0.01, ^***^P < 0.001. LPS-BPH *Porphyromonas gingivalis* lipopolysaccharide induced BPH group, *P.g*-BPH *Porphyromonas gingivalis* induced BPH group, T-BPH Testosterone-induced BPH group, HE hematoxylin and eosin, SM smooth muscle, CF collagen fibers

To investigate whether *P. gingivalis* in the prostate can promote BPH, bacterial transplantation was performed by injecting *P. gingivalis* or *P.g*-LPS into the ventral lobe of the prostate (Fig. 3a). As shown in Fig. 3b, the prostate and seminal vesicles volumes were increased in the T-BPH, LPS-BPH and *P.g*-BPH groups compared with the control. The prostate weight index of the T-BPH group was significantly higher than that of other groups (Fig. 3c). Histologically, prostates taken from the testosterone-induced BPH group, the *P.g*-LPS and the *P. gingivalis* induced groups all displayed increased thickness of the epithelial layer with numerous folds in the prostatic lumen thereby decreasing the volume of the lumen; Masson’s trichrome staining showed that injecting rats with *P. gingivalis* is more likely to cause prostate stromal hyperplasia, but the enlargement of epithelial cells is still obvious (Fig. 3d). Quantitative analysis of histological components showed that the epithelium in LPS-BPH, *P.g*-BPH and T-BPH groups was 3.08-, 2.97- and 3.60-fold compared with the control group, respectively (Fig. 3e). The SM and CF increased in the LPS-BPH, *P.g*-BPH and T-BPH groups, with the SM area was 1.69-, 1.50-, 1.66-fold, and the CF area was 5.02-, 3.81-, 3.87-fold compared with the control group, respectively. The *P.g*-BPH group demonstrated the most obvious statistical effect (*P* < 0.01, Fig. 3f).

### *P. gingivalis* disrupt the balance of cell proliferation and apoptosis in the prostate

**Fig. 4:**
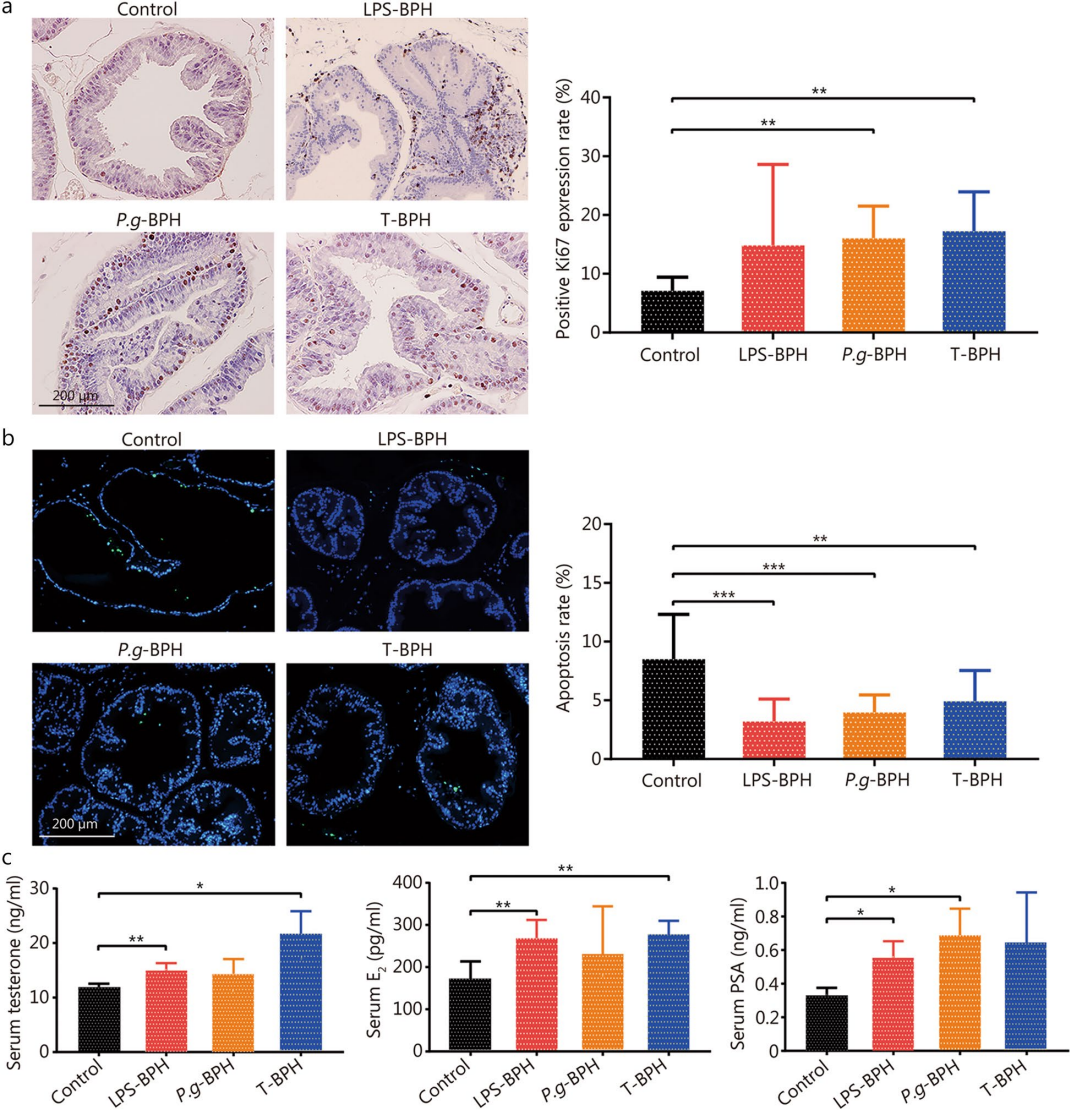
Effects of *P. gingivalis* and *P. gingivalis* LPS on cell proliferation and apoptosis of prostates. **a** Representative image and quantitative analysis histograms of Ki67 immunohistochemical staining for ventral prostates in control, *P.g*-BPH, LPS and T-BPH groups (original magnification ×200). **b** Representative images of TUNEL staining and apoptosis rate (%) histogram of TUNEL positive cells for ventral prostates in control, *P.g*-BPH, LPS and T-BPH groups (original magnification ×200). **c** Enzyme-linked immunosorbent assay (ELISA) for detecting the serum levels of testosterone, E_2_, and PSA. Data are expressed as mean ± SD. ^*^P < 0.05, ^**^P < 0.01, ^***^P < 0.001. LPS-BPH *Porphyromonas gingivalis* lipopolysaccharide induced BPH group, *P.g*-BPH *Porphyromonas gingivalis* induced BPH group, T-BPH Testosterone-induced BPH group, TUNEL terminal deoxynucleotidyl transferase-mediated dUTP nick end labeling, E_2_ estradiol, PSA prostate-specific antigen

Immunohistochemical staining results of Ki67 showed that the positive rate of Ki67 in the prostate tissues of the *P.g*-BPH and the T-BPH group was significantly higher than that of control group (*P* < 0.05), and there was no significant difference between the two groups (Fig. 4a). The results of rat prostate TUNEL staining showed that compared with the control group, the prostate epithelial cells in the LPS-BPH, *P.g*-BPH and T-BPH groups increased significantly and protruded into the cavity, while the apoptosis rate was significantly reduced (Fig. 4b). These results indicate that *P. gingivalis* promoted BPH by promoting the proliferation of prostate cells, inhibiting cell apoptosis, and then destroying the balance between the proliferation and apoptosis of prostate cells.

### Effect of *P. gingivalis* on serum levels of testosterone, E_2_ and PSA in rats

Our results showed that the serum levels of testosterone, E_2_ and PSA were higher in the T-BPH group by 1.79-, 1.61- and 1.92-fold compared with the control group, suggesting testosterone administration boosted serum levels of these BPH indicators. The serum concentrations of testosterone and E_2_ were significantly elevated in LPS-BPH and T-BPH groups when compared to control group (Fig. 4c), suggesting serum hormone markers as indicators of prostatic hypertrophy. The serum PSA level in the *P.g*-BPH, LPS-BPH and T-BPH groups were higher than that of control group, but only the *P.g*-BPH and LPS-BPH group had a significant difference (Fig. 4c), suggesting PSA may be characterized as an inflammatory marker in BPH development.

### *P. gingivalis* increases the expression of inflammatory cytokines in prostate tissue

**Fig. 5:**
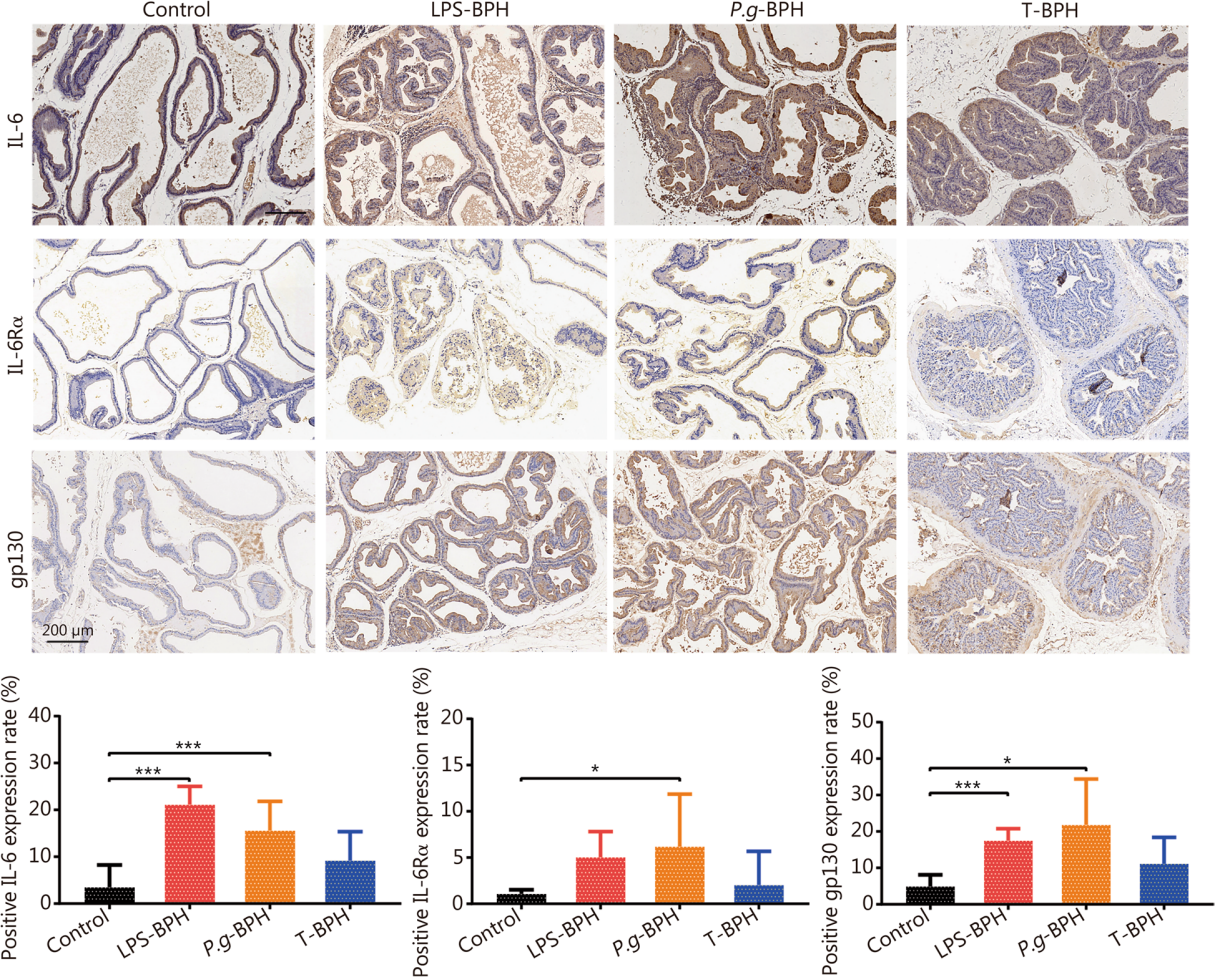
Effects of *P. gingivalis* and *P. gingivalis* LPS on the expression of proinflammatory cytokines in rat prostate tissues. Representative images and quantitative analysis histograms of IL-6, IL-6α and gp130 immunohistochemical staining in prostate tissue of rats in control, *P.g*-BPH, LPS and T-BPH groups (original magnification ×200). Data are expressed as mean ± SD. ^*^P < 0.05, ^***^P < 0.001. IL-6 interleukin-6, IL-6Rα interleukin-6 receptor-α, LPS-BPH *Porphyromonas gingivalis* lipopolysaccharide induced BPH group, *P.g*-BPH *Porphyromonas gingivalis* induced BPH group, T-BPH testosterone-induced BPH group

The results of HE staining showed that a large number of inflammatory cells were infiltrated in the prostate tissue of the *P.g*-BPH group. To further investigate the possible mechanism of *P. gingivalis*-induced BPH symptoms, immunohistochemical staining for interleukin-6 (IL-6), IL-6Rα and gp130 was carried out in the prostate tissues, as shown in Fig. 5. The rates of IL-6 positive cells in both *P.g*-BPH, LPS-BPH and T-BPH groups were higher than that of control group by 4.47-, 6.06- and 2.63-fold, but only the *P.g*-BPH and LPS-BPH group showed a significant difference (*P* < 0.001). The number of IL-6Rα positive cells in *P.g*-BPH group (5.74-fold), and the number of gp130 positive cells in LPS-BPH group (3.57-fold) and *P.g*-BPH group (4.47-fold) were significantly higher than those in control group (*P* < 0.05 or *P* < 0.001). These results indicated the injection of the prostate with *P. gingivalis* promoted the expression of pro-inflammatory cytokines, and it may play a role through IL-6 and its receptors.

### *P. gingivalis* may promote BPH by inducing the imbalance of proliferation and apoptosis of prostate cells through the IL-6/IL-6Rα/Akt signaling pathway

**Fig. 6:**
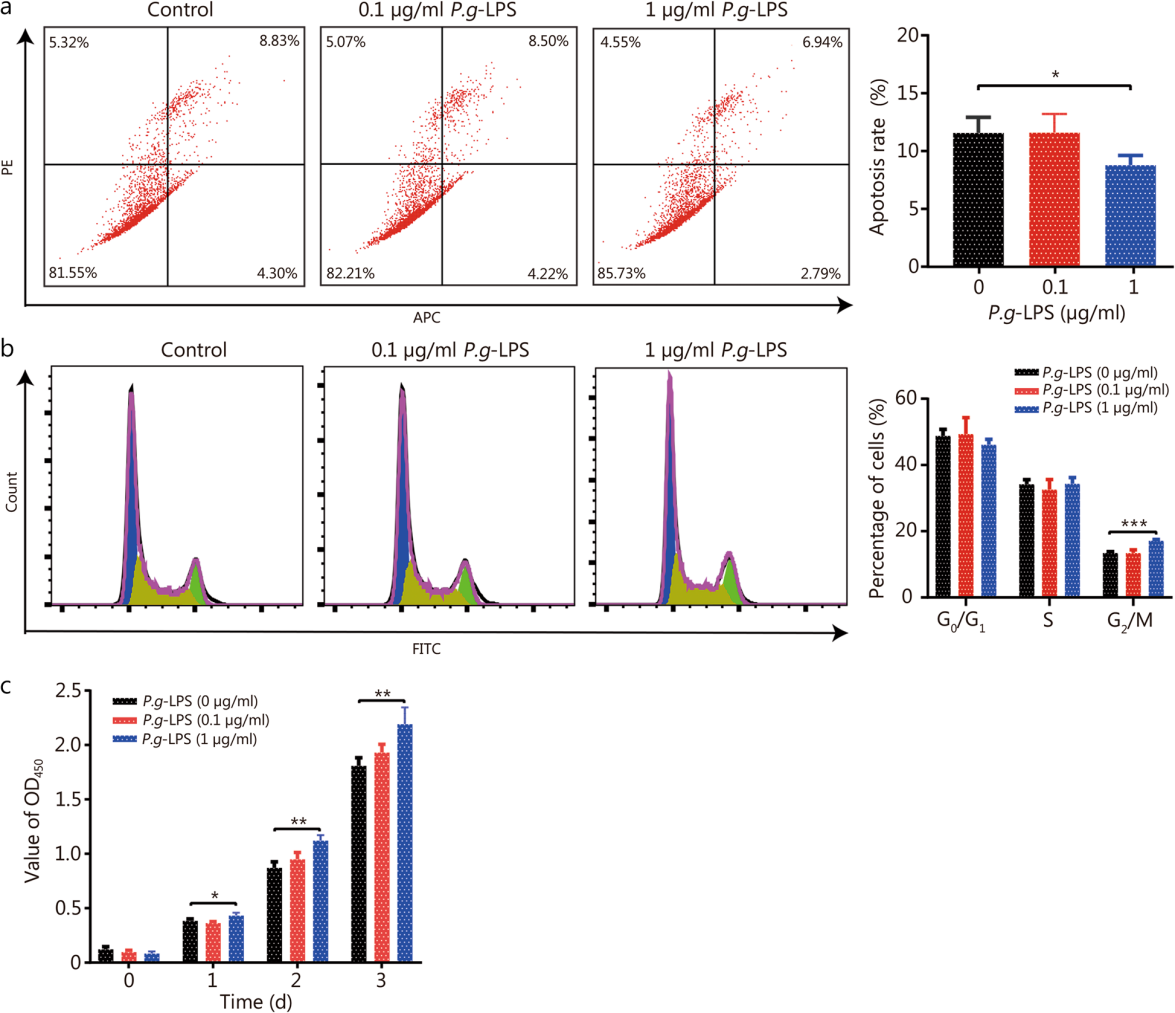
*P. gingivalis* LPS decreased apoptosis of WPMY-1 cells and increased cell growth. **a** Flow cytometry apoptotic representative images and apoptosis rate histogram of WPMY-1 cells treated with selected concentrations of 0, 0.1 and 1 μg/ml *P.g-*LPS for 24 h. **b** Flow cytometry representative images and quantitative analyses of cell cycle of WPMY-1 cells treated with selected concentrations of 0, 0.1 and 1 μg/ml *P.g-*LPS for 24 h. **c** Cell viability of WPMY-1 cells treated with 0, 0.1 and 1 μg/ml *P.g-*LPS at different time points of 0, 24, 48 and 72 h by CCK-8 assay. Data are expressed as mean ± SD. ^*^P < 0.05, ^**^P < 0.01, ^***^P < 0.001. LPS lipopolysaccharide, PE phycoerythrin, APC Allophycocyanin, FITC fluoresceinisothiocyanate

The above findings suggest that *P. gingivalis* may play an important role in the progression of BPH and may play a role through inflammatory cytokines. To further investigate the possible mechanism, different concentrations of *P.g*-LPS were used to treat WPMY-1 cells. *P.g*-LPS treatment at 1 µg/ml for 24 h significantly decreased cell apoptosis, promoted cell mitosis and cell proliferation as compared to the untreated control, and no change was observed at 0.1 µg/ml (Fig. 6), suggesting that *P.g*-LPS could induce the imbalance of cell proliferation and apoptosis. When the concentration of *P.g*-LPS was 10 μg/ml, it showed similar effects in inhibiting cell apoptosis as compared to 1 μg/ml, whereas it had no effect on cell cycles (Additional file 1: Fig. S4). Thus, a 1 µg/ml concentration of *P.g*-LPS was used for further experiments.

**Fig. 7:**
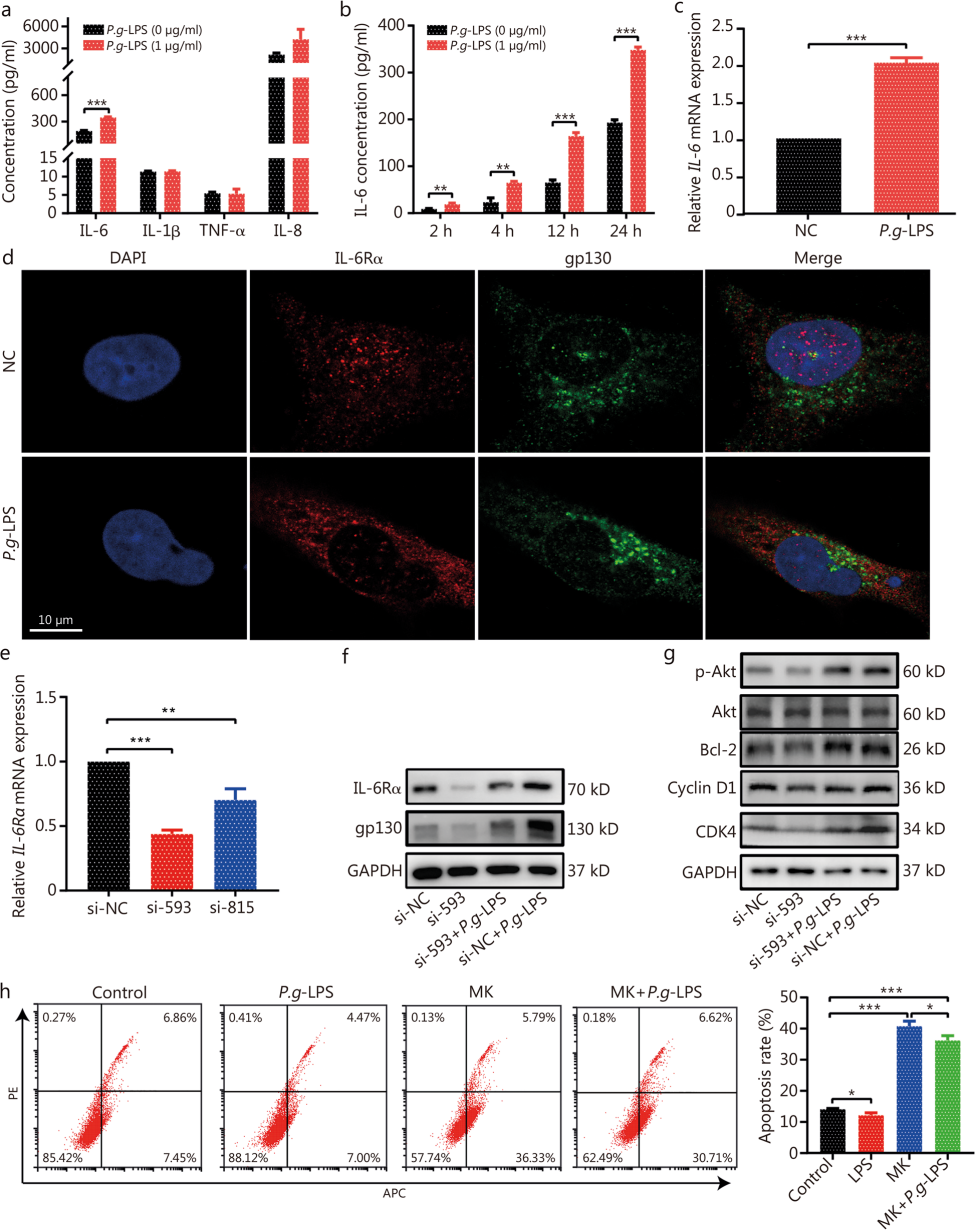
*P. gingivalis* LPS may regulate IL-6/IL-6R/Akt signaling pathway. **a** The concentration of inflammation cytokines in WPMY-1 cells untreated or treated with 1 μg/ml *P.g-*LPS for 24 h. **b** The concentration of IL-6 in WPMY-1 cells untreated or treated with 1 μg/ml *P.g-*LPS at different time points of 2, 4, 12, and 24 h. **c** The expression *IL-6* mRNA in WPMY-1 cells untreated or treated with 1 μg/ml *P.g-*LPS for 24 h. **d** Immunofluorescence staining for IL-6Rα (red) and gp130 (green) in WPMY-1 cells untreated or treated with 1 μg/ml *P.g-*LPS for 4 h (original magnification ×100). **e** Knockdown of *IL-6Rα* was assessed by qPCR. **f** Western blotting analysis of IL-6R and gp130 proteins expression upon indicated treatment. **g** Western blotting analysis of Akt signaling proteins upon indicated treatment. **h** Flow cytometry analyses for apoptosis of WPMY-1 cells treated MK (p-Akt inhibitor) or MK combined with *P.g-*LPS. Data are expressed as mean ± SD. ^*^P < 0.05, ^**^P < 0.01, ^***^P < 0.001. LPS lipopolysaccharide, IL-6 interleukin-6, IL-6Rα interleukin-6 receptor-α

Subsequently, WPMY-1 cells were treated with 1 μg/ml *P.g*-LPS for 24 h, the concentration of inflammatory cytokines IL-6, interleukin-1β (IL-1β), tumor necrosis factor-α (TNF-α) and interleukin-8 (IL-8) were detected. Compared with the control group, *P.g*-LPS treatment significantly increased the concentration of IL-6 (Fig. 7a), and it increased the IL-6 in a time-dependent manner (Fig. 7b). We also found that *P.g*-LPS treatment up-regulated *IL-6* mRNA expression (Fig. 7c). Immunofluorescent staining showed that IL-6Rα and gp130 were expressed in both cell membrane and cytoplasm (Fig. 7d). To further confirm whether IL-6 acts through the IL-6/IL-6Rα/gp130 complex, we steadily knocked down *IL-6Rα* in WPMY-1 cells by two independent IL-6Rα siRNAs, and selected si-593, which had a better interference effect, for follow-up experiments (Fig. 7e). Knockdown of *IL-6Rα* decreased the protein level of IL-6Rα and gp130, additionally *P.g*-LPS partially abrogated the IL-6Rα knockdown-caused downregulation of IL-6Rα and gp130 protein (Fig. 7f). In terms of intracellular signaling, we found that *P.g*-LPS significantly promoted Akt phosphorylation, B cell lymphoma-2 (Bcl-2) and cyclin-dependent kinase 4 (CDK4) protein level. IL-6Rα knockout significantly inhibited Akt phosphorylation, Bcl-2 and CDK4 protein level, and *IL-6Rα* knockout partially reversed the activation of Akt phosphorylation and upregulated CDK4 by *P.g*-LPS (Fig. 7g). Further treatment of prostate cells with MK (an inhibitor of p-Akt), significantly promoted apoptosis, and *P.g*-LPS could partially reverse its effect (Fig. 7h). The above results suggest that *P.g*-LPS activates the Akt pathway through the IL-6/IL-6R/gp130 complex, which destroys the balance of prostate cell proliferation and apoptosis and induces BPH.

## Discussion

Observational studies have shown that after adjusting for confounding factors, medical history data, and laboratory data, BPH is significantly associated with periodontitis, and the odds ratios are all greater than 1.5 [9, 12, 35]. Elimination of periodontal pathogens through appropriate periodontal therapy can reduce the clinical symptom score of the prostate, but the mechanism is unknown [14]. Therefore, it is crucial to determine the link between the two diseases and the possible mechanism for further research. Estemalik et al. [28] indicated that at least one oral bacterial DNA was detected in the Pf of 90% (9/10) patients with BPH, suggesting that the transfer of oral pathogenic bacteria may be a linking factor between periodontitis and BPH. However, it was not clear which pathogens played a role, and there was no evidence that oral pathogens are viable at the prostate site. In this study, we found that the distal metastasis of oral pathogen *P. gingivalis* may be a mechanism involved in periodontitis promoted BPH. *P. gingivalis* may be involved in the progression of BPH through the IL-6/IL-6Rα/Akt signaling pathways.

Our findings provide evidence for a previously unknown mechanism, namely the relationship between periodontitis and BPH. Periodontitis patients introduce oral microbes into their blood multiple times a day by perturbation of periodontal tissues (e.g., toothbrushes) [36]. It has been reported that periodontal pathogens may be transferred from infected periodontal membranes to atherosclerosis, joint synovial fluid, placenta and other distal tissues and initiate metastatic infection after colonization [37–40]. In this study, we isolated live bacteria through microbial culture and identified their genomes by 16S rDNA sequencing, demonstrating the presence of viable oral pathogenic bacteria in the prostate, and the abundance of *P. gingivalis* was the highest. *P. gingivalis* is the most important pathogen associated with periodontitis, and it is a red complex bacterium with the ability to bind to erythrocytes and penetrate epithelial tissues [5]. Active and invasive *P. gingivalis* have been detected in human atherosclerotic plaque tissue and co-localization has been observed [23, 41]. Given the characteristics of *P. gingivalis*, our observations in the prostate are also plausible and consistent with the previous report [28]. Further through animal experiments, we found that periodontitis significantly promoted testosterone-induced hyperplasia which is indicated by the aggravation of the pathological structure alterations in the composite model, and *P. gingivalis* may have played a key role in hyperplasia and aggravation. Then the transplantation of *P. gingivalis* into the prostate can induce prostatic hyperplasia and collagen fibrosis, which is similar to the symptoms of BPH induced by testosterone propionate. BPH is a chronic disease with slow progression. On the one hand, samples were collected after 4 weeks of experimentation, the experiment time was short. On the other hand, *P. gingivalis* infection mainly consists of interstitial hyperplasia, the prostate weight index of the *P.g*-BPH and LPS-BPH group was not enough to observe significant changes. The obvious symptoms of prostatic hyperplasia induced by *P. gingivalis* suggest that it can promote the progression of BPH. Studies have shown that BPH manifestation is associated with elevated testosterone and inflammation [14, 42]. A positive correlation between elevated serum E_2_ levels and the development of BPH has also been reported [43]. Also, prostatic inflammation contributes greatly to elevated serum levels of PSA [14]. Our results were consistent with these studies, and identified the above serum markers indicative of prostatic hypertrophy and inflammation.

The imbalance between cell proliferation and apoptosis leads to continuous growth of prostate cells and is a pathogenic mechanism of BPH [3, 44]. Inflammation, which is known to lead to increased cell proliferation and decreased cell apoptosis, has been shown to be one of the factors in testosterone-induced BPH [45, 46]. Several studies have shown that the expression of inflammatory cytokines IL-6 is significantly up-regulated in patients with BPH, and animal experiments suggest that IL-6 plays an important role in the progression of BPH [47, 48]. *P. gingivalis* periodontal infection may cause cognitive impairment or neuroinflammation via the release of the pro-inflammatory cytokines IL-6, TNF-α, and IL-1β [49, 50]. *P. gingivalis* invasion of cells can activate inflammatory cytokines to induce inflammation [51]. Our study also found that the levels of inflammatory cytokines IL-6 and its receptors (IL-6Rα and gp130) increased in the prostate tissue of *P. gingivalis*-transplanted rats. When 1 µg/ml *P.g*-LPS was used to infect cells, IL-6 expression was significantly increased. The proliferation of prostatic cells was correlated with IL-6, which was consistent with the previous results [52, 53]. IL-6 activates molecules that are involved in intracellular signal transduction, such as Janus kinases (JAKs), signal transducer and activator of transcription 3 (STAT3), mitogen-activated protein kinases (MAPK) [54, 55]. *P. gingivalis* can mediate intracellular signaling, such as affecting cell apoptosis through PI3K/Akt, JAK/STAT and other pathways [56]. It has been reported that the PI3K/Akt signaling pathway is involved in the regulation of cell proliferation, survival and apoptosis and the progression of BPH through downstream effectors such as Bcl-2 [57, 58]. Together, we have found in this study that both IL-6Rα and gp130 were expressed on the membrane of prostate cells, and IL-6 affected cell function by activating the intracellular Akt signaling pathway through the classical signaling system IL-6/IL-6Rα/gp130 complex. *P.g*-LPS partially reversed the proapoptotic effect of p-Akt inhibitors on prostate cells. LPS is the major inflammatory mediator for gram negative bacteria, and is able to elicit cell inflammatory responses via interaction with Toll like receptors (TLRs) [59]. Previous studies have demonstrated *E. coli*-LPS as a key factor in the TLR4/TGF-β1 signaling pathway for induction of epithelial mesenchymal transition in BPH-1 cells, thus resulting in BPH development [60]. Prostate smooth muscle cells are strongly involved in the development and progression of BPH, they are also capable of responding to LPS, with the TLR4 signaling pathway being involved in this response [61]. *P.g*-LPS induces cognitive dysfunction, mediated by neuronal inflammation via activation of the TLR4 signaling pathway in mice [50]. The receptor for advanced glycation end products can interact with *P.g*-LPS, and this receptor can be responsible for microglial activation and production of proinflammatory mediators in Alzheimer’s disease, which is prevalent among elderly men worldwide [62]. Thus, further investigation is warranted into whether TLRs or other pathways involved in the proliferation and apoptosis imbalance of prostate cells induced by *P.g*-LPS.

This study has certain limitations. First, our clinical sample size is limited and there is no classification based on disease severity. A well-controlled longitudinal study with a larger sample size is required to clarify the relationship between periodontitis and BPH by comparing the bacterial flora between different organs and groups of disease severity. Second, sequencing after culture can detect live bacteria in the prostate, but some bacteria that are difficult to culture may be missed. DNA was extracted directly from specimens and sequenced by 16S rRNA or metagenomics which could detect *P. gingivalis* gene fragments in the arterial wall to determine colonization. However these techniques are unable to assess the activity status and reproduction of *P. gingivalis* after colonization or to determine whether the gene fragments are from intact bacteria. So, we cultured the samples. Indeed, although we use three kinds of culture plates for bacterial culture, there are some bacteria that are not easy to culture, and the collected bacterial growth colonies cannot contain all the bacteria in the sample. However, the purpose of sequencing after culture is to determine the survival status of bacteria. And the purpose of this study is not to detect all microorganisms, but to sequence in order to select the microorganisms that may play a role, and verify these through experiments. More potential microbes will be studied in combination with new sequencing data in the future. Third, by what route does *P. gingivalis* reach the prostate has not been explored in this study. Previous studies have shown that periodontal bacteria can disseminate by different routes–haematogenous, oro-pharyngeal and oro-digestive to reach extra-oral sites where they can cause or exacerbate inflammatory pathologies [5, 14]. Active and invasive *P. gingivalis* were detected in atherosclerotic cardiovascular and colorectal cancer tissues, which have the ability to bind to red blood cells and penetrate epithelial tissue [23, 60]. We have assumed that *P. gingivalis* may reach the prostate through blood or digestive system, but the specific route needs to be further determined. Additionally, the urine test data indicative of prostatic hypertrophy, such as urine volume and micturition frequency should be recorded for each individual animal in future studies.

## Conclusions

**Fig. 8:**
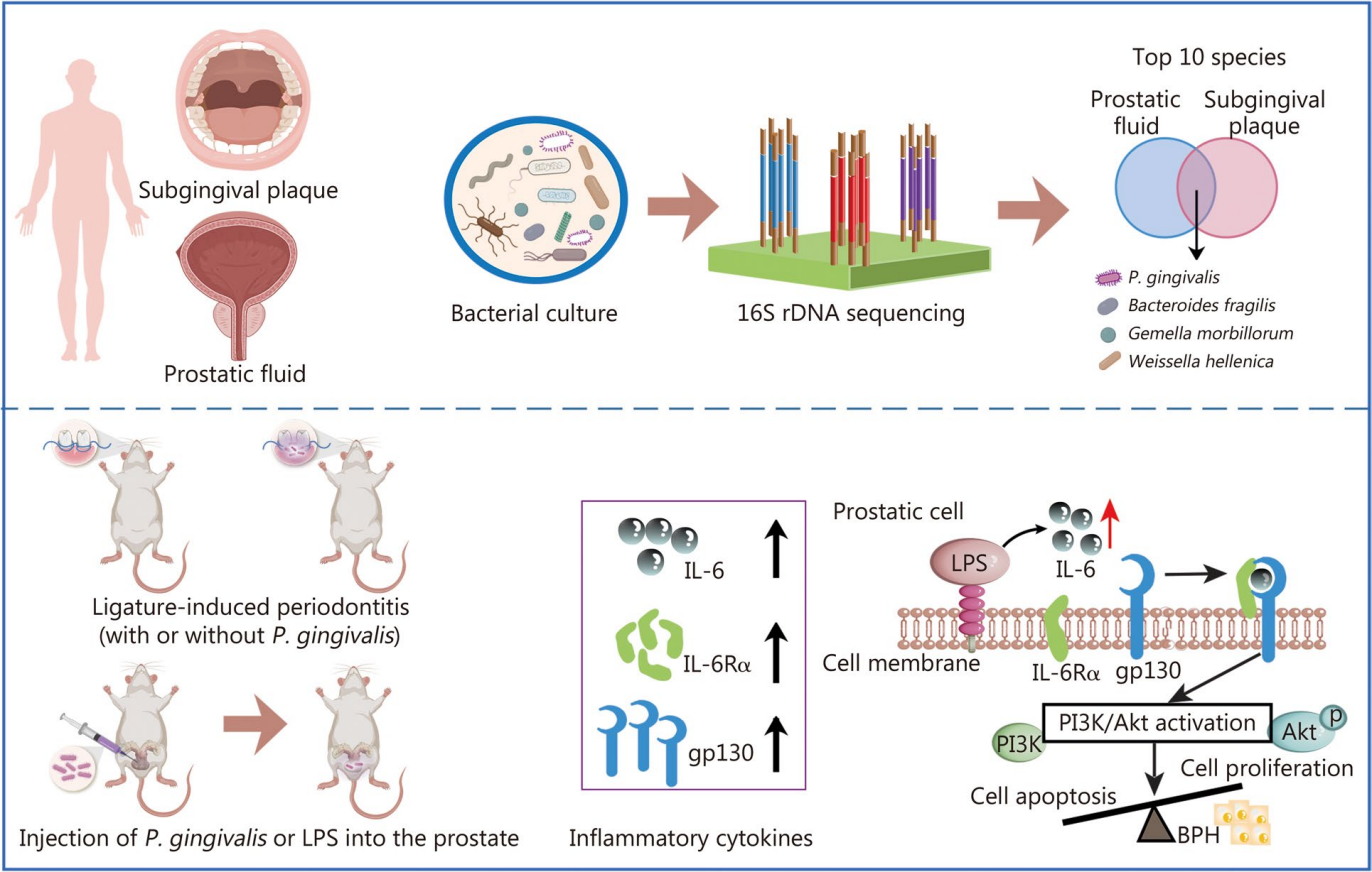
Schematic diagram showing the mechanisms of *P. gingivalis* exacerbates BPH via IL-6/IL-6R pathway. BPH benign prostatic hyperplasia, *P. gingivalis Porphyromonas gingivalis*, LPS lipopolysaccharide, IL-6 interleukin-6, IL-6Rα interleukin-6 receptor-α

In conclusion, we found in the present study that relative high abundance of *P. gingivalis* was detected in the prostate of BPH patients, and animal experiments also confirmed *P. gingivalis* as an inducer of BPH. *P. gingivalis* may affect the progression of BPH through IL-6/IL-6R inflammation and Akt signaling pathways, suggesting that the distal metastasis of oral pathogens is a possible mechanism for periodontitis to promote BPH (Fig. 8). Although there are other oral pathogens, their abundance is low, so this study mainly focused on the role of *P. gingivalis*. *P.g*-LPS is an important virulence factor of *P. gingivalis*, and it is confirmed by animal experiments that both *P. gingivalis* and *P.g*-LPS can promote BPH. The role of other virulence factors of *P. gingivalis* will be detected simultaneously while further deepening knowledge of the mechanism of *P. gingivalis* on BPH. This study provides evidence and research direction for the development of new prevention and treatment strategies for BPH from the perspective of periodontal health intervention in the future.

### Supplementary Information

**Additional file 1: Table S1** List of antibodies used for immunofluorescence and immunohistochemistry staining. **Table S2** Primers and RNA oligonucleotides sequences used in this study. **Table S3** List of antibodies used for western blot analysis. **Table S4** Top 10 species of subgingival plaque and prostatic fluid. Table S5 Detection of oral pathogens in prostatic fluid and subgingival plaque of each patient. **Fig. S1** Relative abundances of microbial composition at the family level. **Fig. S2** Histogram of relative abundance of microbial composition at the genus level in subgingival plaque and prostatic fluid of 8 patients. **Fig. S3** Alveolar bone loss and histological changes in rat periodontal tissues. **Fig. S4** Flow cytometry analyses for apoptosis and cell cycle of WPMY-1 cells treated with selected concentrations of *P. gingivalis* LPS.

## Data Availability

The 16S rDNA microbiome sequencing data that support the findings of this study are openly available in NCBI BioProject at: https://www.ncbi.nlm.nih.gov/bioproject, reference number: PRJNA863440. The other data used to support the findings of this study are included within the article.

## Abbreviations

ANOVA: Analysis of variance
BPH: Benign prostatic hyperplasia
Bcl-2: B cell lymphoma-2
CCK-8: Cell Counting Kit-8 assay
CCS: Circular consensus sequence
CDK4: Cyclin-dependent kinase 4
CF: Collagen fibers
CEJ-ABC: Cement-enamel junction to alveolar bone crest
EP: Iigature-induced experimental periodontitis group
Epithelial: Prostate epithelial
EP + BPH: Composite group of EP and BPH
E_2_: Estradiol
*E. coli*: *Escherichia coli*
gp130: Glycoprotein 130
HE: Hematoxylin and eosin
IF: Immunofluorescence
IHC: Immunohistochemistry
IL-1β: Interleukin-1β
IL-6: Interleukin-6
IL-6R: Interleukin-6 receptor
IL-6Rα: Interleukin-6 receptor α
IL-8: Interleukin-8
JAK: Janus kinase
LPS-BPH: *Porphyromonas gingivalis* lipopolysaccharide induced BPH group
Micro-CT: Micro-computed tomographic
MAPK: Mitogen-activated protein kinases
OTUs: Operational taxonomic units
*P. gingivalis*: *Porphyromonas gingivalis*
*P.g*: *P. gingivalis* induced periodontitis group
*P.g*-BPH: *Porphyromonas gingivalis* induced BPH group
*P.g*-LPS: *Porphyromonas gingivalis* lipopolysaccharide
p-Akt: Phosphorylated Akt
PCA: Principal component analysis
PCoA: Principal co-ordinate analysis
PSA: Prostate-specific antigen
Pf: Prostatic fluid
RDP: Ribosomal Database Project
RT-qPCR: Reverse transcription-quantitative PCR
SM: Smooth muscle
STAT: Signal transducer and activator of transcription
Sp: Subgingival plaque
T-BPH: Testosterone-induced BPH group
TLRs: Toll like receptors
TNF-α: Tumor necrosis factor-α
TUNEL: Terminal deoxynucleotidyl transferase-mediated dUTP nick end labeling
