# Supplementary material for: P. gingivalis in oral-prostate axis exacerbates benign prostatic hyperplasia via IL-6/IL-6R pathway
Source: Mil Med Res. 2024 May 20;11:30. doi: 10.1186/s40779-024-00533-8 (PMC11103868; doi:10.1186/s40779-024-00533-8)
Supplement: Supplementary file 1 — Additional file 1: Table S1 List of antibodies used for immunofluorescence and immunohistochemistry staining. Table S2 Primers and RNA oligonucleotides sequences used in this study. Table S3 List of antibodies used for western blot analysis. Table S4 Top 10 species of subgingival plaque and prostatic fluid. Table S5 Detection of oral pathogens in prostatic fluid and subgingival plaque of each patient. Fig. S1 Relative abundances of microbial composition at the family level. Fig. S2 Histogram of relative abundance of microbial composition at the genus level in subgingival plaque and prostatic fluid of 8 patients. Fig. S3 Alveolar bone loss and histological changes in rat periodontal tissues. Fig. S4 Flow cytometry analyses for apoptosis and cell cycle of WPMY-1 cells treated with selected concentrations of P. gingivalis LPS. [file 40779_2024_533_MOESM1_ESM.pdf]

Table S1 Antibodies used for immunofluorescence (IF) and immunohistochemistry staining (IHC)

Antibodies	Source species	Dilution ratio*	Supplier
**Primary antibody**			
Ki67	Rabbit	1:500 (IHC)	Servicebioe, GB111141
IL-6	Rabbit	1:100 (IF/IHC)	Abclonal, A0286
IL-6Rα	Mouse	1:100 (IF/IHC)	Santa Cruz, sc-373708
gp130	Rabbit	1:100 (IF/IHC)	Abclonal, A14656
**Secondary antibody**			
Anti-rabbit IgG H&L (Alexa Fluor® 555)	Goat	1:1000 (IF)	Abacm, ab150078
Anti-mouse IgG H&L (Alexa Fluor® 647)	Goat	1:1000 (IF)	Abacm, ab150115
Anti-mouse-IgG-HRP	Goat	1:200 (IHC)	Servicebio, GB23301
Anti-rabbit-IgG-HRP	Goat	1:200 (IHC)	Servicebio, GB23303

*Primary antibody dilution buffer for IF, Beyotime Biotechnology (P0023A); Primary antibody dilution buffer for IHC, Servicebio (G20250); Secondary antibody dilution buffer for IF, PBST; Secondary antibody dilution buffer for IHC, PBS Solution. *IL-6* interleukin-6, *IL-6Rα* interleukin-6 receptor-α

Table S2 Primers and RNA oligonucleotides sequences used in this study.

Gene (human)	Forward primer (5' – 3')	Reverse primer (5' – 3')
IL-6	ACCTTCCAAAGATGGCTGAA	GGCTTGTTCCTCACTACTCTCAA
IL-6Rα	ACTTGCTGGTGGATGTTCCC	AGCCTTTGTCGTCAGGGATG
si IL-6Rα-593	GGAAGACAAUGCCACUGUUTT	AACAGUGGCAUUGUCUUCCTT
si IL-6Rα-815	CCUCAGCAAUGUUGUUUGUTT	ACAAACAACAUUGCUGAGGTT
β-actin	CCACACCCGCCACCAGTTC	GACCCATTCCCACCATCACACC

IL-6 interleukin-6, *IL-6Rα* interleukin-6 receptor-α

Table S3 Antibodies used for Western blotting analysis

Antibodies	Source species	Dilution ratio*	Supplier
**Primary antibody**			
IL-6Rα	Rabbit	1:1000	Santa Cruz, sc-373708
gp130	Rabbit	1:1000	Abcam, ab283685
Akt	Mouse	1:1000	Santa Cruz, sc-81434
p-Akt	Rabbit	1:2000	Santa Cruz, sc-514032
Cyclin D1	Rabbit	1:1000	Abcam, ab16663
CDK4	Mouse	1:1000	Santa Cruz, sc-23896
Bcl-2	Rabbit	1:1000	Abcam, ab196495
GAPDH	Rabbit	1:10000	Abcam, ab181602
**Secondary antibody**			
Anti-mouse-IgG (H + L)-HRP	Goat	1:10000	Beyotime Biotechnology, A0216
Anti-rabbit-IgG (H + L)-HRP	Goat	1:10000	Beyotime Biotechnology, A0208

*Primary antibody dilution buffer, Beyotime Biotechnology (P0023A); Secondary antibody dilution buffer, 1× TBST (Solarbio, T1081). *IL-6Rα* interleukin-6 receptor-α

Table S4 Top 10 species of subgingival plaque (Sp) and prostatic fluid (Pf)

Sp		Prostatic fluid	
Top 10 species	Mean relative abundance	Top 10 species	Mean relative abundance
s__*Porphyromonas gingivalis*	33.89%	s__*Escherichia fergusonii*	13.83%
s__*Bacteroides fragilis*	26.79%	s__*Pseudomonas aeruginosa*	12.42%
s__*Gemella morbillorum*	13.03%	s__*Weissella hellenica*	11.95%
s__*Capnocytophaga ochracea*	4.10%	s__*Porphyromonas gingivalis*	7.31%
s__*Veillonella parvula*	2.88%	s__*Bacteroides fragilis*	5.38%
s__*Staphylococcus epidermidis*	1.93%	s__*Lactobacillus plantarum*	1.58%
s__*Weissella hellenica*	1.64%	s__*Lactobacillus leichmannii*	0.90%
s__*Granulicatella adiacens*	1.11%	s__*Streptococcus oralis*	0.73%
s__*Parvimonas micra*	1.11%	s__*Enterococcus mundtii*	0.65%
s__*Prevotella loescheii*	0.91%	s__*Gemella morbillorum*	0.63%

Table S5 Detection of oral pathogens in subgingival plaque (Sp) and prostatic fluid (Pf) of each patient

Patients	*Porphyromonas gingivalis*		*Bacteroides fragiliss*		*Capnocytophaga ochracea*		*Parvimonas micra*		*Streptococcus oralis*	
	Sp	Pf	Sp	Pf	Sp	Pf	Sp	Pf	Sp	Pf
1	-	-	+	+	-	-	-	-	-	-
2	+	+	+	+	-	+	+	+	+	-
3	+	+	-	+	+	-	-	-	-	+
4	+	+	-	+	-	+	-	+	-	+
5	+	+	-	+	-	+	-	+	-	+
6	-	-	-	+	+	+	+	-	-	-
7	+	+	+	+	-	+	-	+	-	-
8	-	-	+	+	-	+	+	-	-	-

"+" indicate the bacteria were detected; "-" indicate the bacteria were not detected

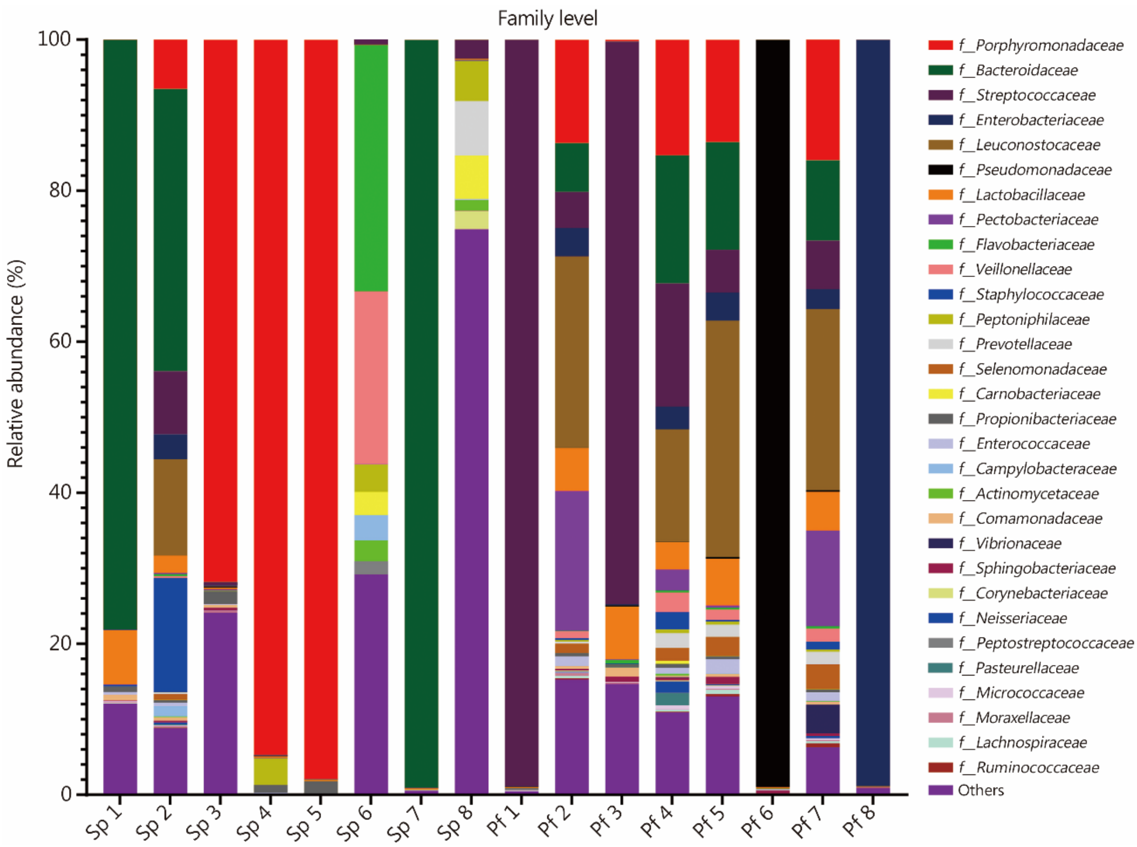

Fig. S1 Relative abundance of microbial composition at the family level in all samples. Each bar represents a subject sample and each colored box represents a bacterial family. Sp subgingival plaque, Pf prostatic fluid

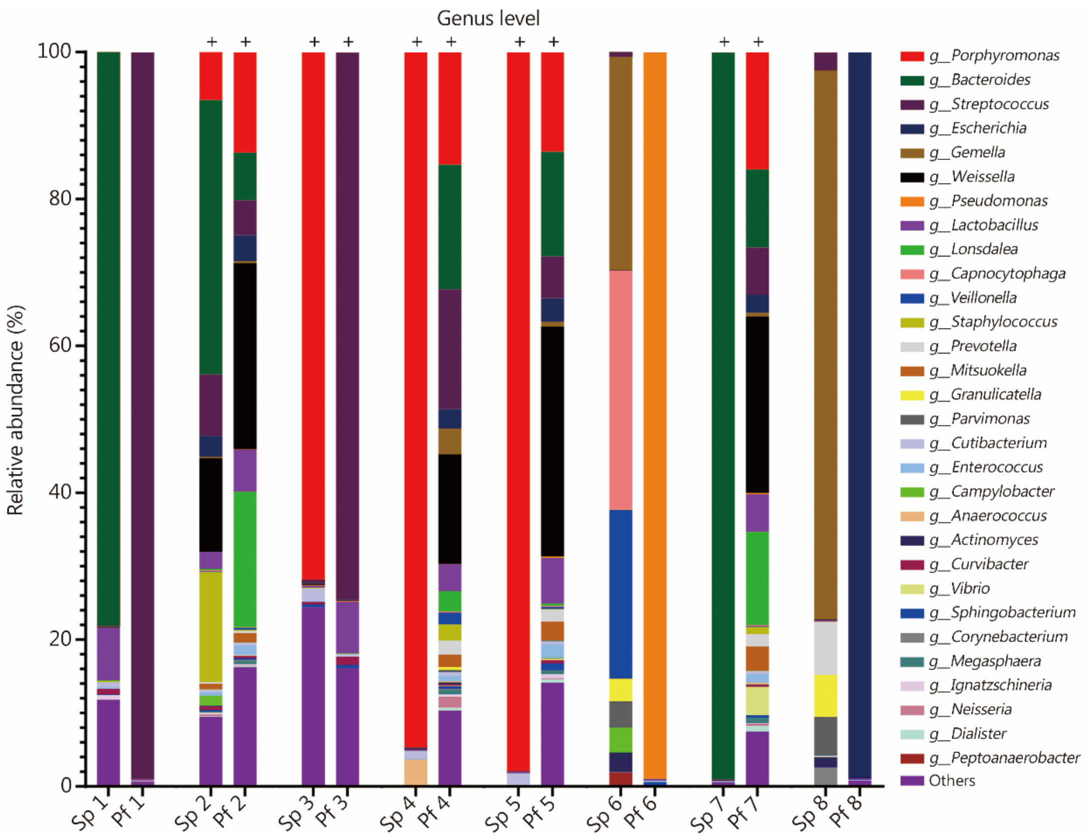

Fig. S2 Histogram of relative abundance of microbial composition at the genus level in subgingival plaque (Sp) and prostatic fluid (Pf) of 8 patients. Each bar represents a subject sample and each colored box represents a bacterial genus. + indicates that *P. gingivalis* was detected

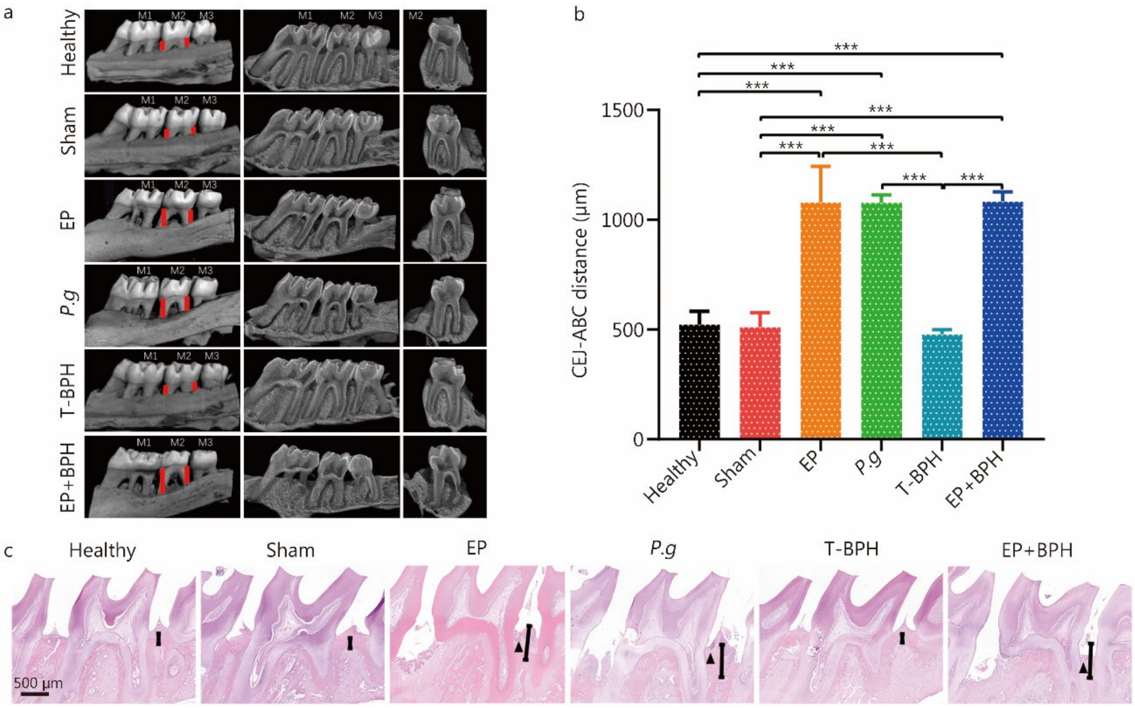

Fig. S3 Alveolar bone loss and histological changes in rat periodontal tissues. **a** Representative image obtained by micro-CT. The red lines showed the linear distance from the cement-enamel junction (CEJ) to the alveolar bone crest (ABC) for the maxillary second molar. **b** Quantitative analysis of the CEJ-ABC distance from the healthy, sham, EP, *P.g*, T-BPH and EP + BPH groups. Data are presented as mean ± SD, $^{***}P < 0.001$. **c** Representative figures from HE staining for the second maxillary alveolar bones (arrows indicate inflammatory changes in the gingival epithelium, scale plates indicate linear distance from CEJ to ABC; original magnification ×40). $^{***}P < 0.001$. EP ligature-induced experimental periodontitis group, *P.g porphyromonas gingivalis* induced BPH group, T-BPH testosterone-induced BPH group, EP+BPH composite group of EP and BPH

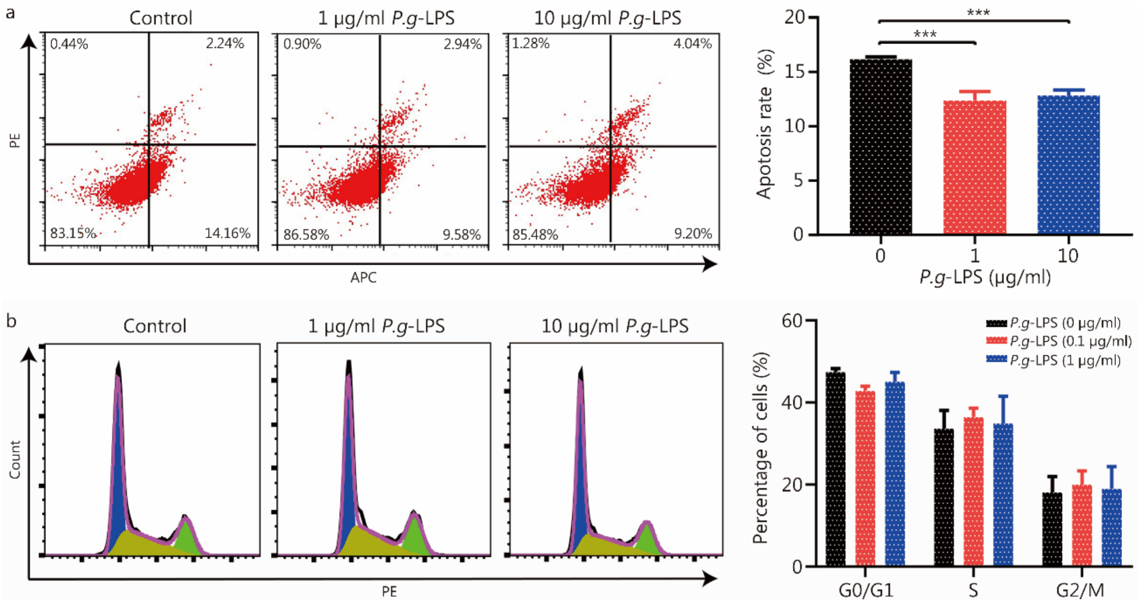

Fig. S4 Flow cytometry analyses for apoptosis and cell cycle of WPMY-1 cells treated with selected concentrations of *P. gingivalis* LPS. **a** Flow cytometry analyses for apoptosis of WPMY-1 cells treated with selected concentrations of 0, 1 and 10 µg/ml *P.g*-LPS for 24 h, respectively. **b** Flow cytometry analyses for cell cycle in WPMY-1 cells treated with selected concentrations of 0, 1 and 10 µg/ml *P.g*-LPS for 24 h, respectively. Data are expressed as mean ± SD. $^{*}P < 0.05$, $^{***}P < 0.001$. *P.g*-LPS *Porphyromonas gingivalis* lipopolysaccharide, PE phycoerythrin, APC allophycocyanin
